# Would government compensation of living kidney donors exploit the poor? An empirical analysis

**DOI:** 10.1371/journal.pone.0205655

**Published:** 2018-11-28

**Authors:** Philip J. Held, Frank McCormick, Glenn M. Chertow, Thomas G. Peters, John P. Roberts

**Affiliations:** 1 Division of Nephrology, Stanford University School of Medicine, Palo Alto, California, United States of America; 2 Independent Researcher, Walnut Creek, California, United States of America; 3 Division of Nephrology, Stanford University School of Medicine, Palo Alto, California, United States of America; 4 Department of Surgery, University of Florida, Jacksonville, Florida, United States of America; 5 Department of Surgery, University of California San Francisco, San Francisco, California, United States of America; University of Toledo, UNITED STATES

## Abstract

Government compensation of kidney donors would likely increase the supply of kidneys and prevent the premature deaths of tens of thousands of patients with kidney failure each year. The major argument against it is that it would exploit the poor who would be more likely to accept the offers of compensation. This overlooks the fact that many poor patients desperately need a kidney transplant and would greatly benefit from an increased supply of kidneys. The objective of this study is to empirically test the hypothesis that government compensation of kidney donors would exploit the poor. Exploitation is defined by economists and several noted ethicists as paying donors less than the fair market value of their kidney. Exploitation is expressed in monetary terms and compared with the economic benefit recipients receive from a transplant. Data are from the Scientific Registry of Transplant Recipients and the United States Renal Data System annual data reports. Educational attainment is used as a proxy for income. We estimate that if the government rewards living donors with a package of non-cash benefits worth $75,000 per kidney, donors would not be exploited. Much more important, this compensation would likely end the kidney shortage, enabling many more patients with kidney failure to obtain transplants and live longer and healthier lives. The value of kidney transplantation to a U.S. recipient is about $1,330,000, which is an order of magnitude greater than any purported exploitation of a living donor (zero to $75,000). Consequently, the aggregate net benefit to the poor alone from kidney transplantation would increase to about $12 billion per year from $1 billion per year currently. Most of the benefit would accrue to poor kidney recipients. But poor donors would receive the fair market value of their kidney, and hence would not be exploited. If the government wanted to ensure that donors also received a net benefit, it could easily do so by increasing the compensation above $75,000 per donor.

## Introduction

Each year about 125,000 U.S. patients are diagnosed with end-stage renal disease (ESRD) [1] and must either undergo life-long dialysis therapy or obtain a kidney transplant. Only about 31,000 of these patients are added to the transplant waiting list each year, and a little more than half that number actually receive transplants because of a serious and growing shortage of transplantable kidneys [2]. Consequently, the number of U.S. patients requiring maintenance dialysis now approaches half a million, and the number on the transplant waiting list has risen to nearly 100,000 [1,2]. Waiting time for a deceased donor kidney has steadily increased to an average of almost five years, and in many regions wait-time now exceeds life expectancy. Most tragically, each year almost 5,000 patients on the waiting list die and another 4,000 are removed because they are considered too sick to undergo the transplant operation [2].

There is a consensus among economists that the main cause of the kidney shortage is the legal prohibition against compensating kidney donors, and that the shortage could be ended if compensation were permitted [3–8]. Moreover, a growing number of physicians and surgeons have concluded that donor compensation should be seriously considered [9–12]. In May 2014, the American Society of Transplant Surgeons and the American Society of Transplantation published a report calling for the removal of donor financial disincentives and the study of positive incentives to increase organ availability [13]. In November 2015, the House of Delegates of the American Medical Association overwhelmingly passed a resolution to seek regulatory relief so that financial incentives could be evaluated in a real-world pilot study [14].

Although many ethicists appear to be against compensation of kidney donors (see Greasley [15], Kerstein [16], Rippon [17], Sandel [18], and Satz [19]), many others are in favor (see Erin and Harris [20], Richards and colleagues [21], Wilkinson [22], Taylor [23], and Fabre [24]). Prof. Wilkinson has provided a useful survey of the ethics of donor compensation [25].

Those opposed to donor compensation argue that poor people would be more likely than others to accept offers of compensation, so the more affluent would end up buying kidneys from the poor, thereby exploiting them. A 2015 letter to the U.S. Secretary of Health and Human Services, signed by many distinguished members of the transplant community, stated that principles adopted by the World Health Organization “drew on decades of global experience which shows that paying for organs inevitably exploits the poor” [26]. Delmonico et al. [27] make similar arguments. Although this criticism is frequently made, opponents of compensation overlook the strong likelihood that government compensation of living kidney donors would increase the supply of kidneys and enable many more patients with ESRD, including those who are poor, to enjoy longer and healthier lives.

## Methods

The objective of this study is to empirically test the hypothesis that government compensation of living kidney donors would exploit the poor. Exploitation is defined and expressed in monetary terms, and is compared with the estimated benefits received by kidney transplant recipients. We have appended four supplements (S1 File…S4 File) to better explain some of our assumptions and calculations.

### Economic benefit of a kidney transplant to a recipient

Held-McCormick et al. [8] have estimated the discounted present value of the economic benefit of a kidney transplant over a recipient’s lifetime in two situations. The first is for current conditions in the U.S. in which donor compensation is prohibited and as a result there is a serious shortage of transplant kidneys. Consequently, transplant recipients are able to obtain, on average, only one transplant, the value of which is approximately $937,000 ([8] Table 3). In the second situation, donors are compensated for the fair market value of a kidney, which would likely end the kidney shortage. With kidneys readily available, transplant recipients would be able to obtain, on average, two transplants, with a total estimated value of $1,330,000 ([8] Table 3). (The average waiting list patient who receives a first transplant has a life expectancy of 19.3 years, but the average graft lasts only 12.6 years. So the average recipient would receive two transplants. Some recipients would receive more and some less.)

### The exploitation of kidney donors

The term exploitation is defined as paying less than the fair market value of the good or service being sold. This definition is well grounded in the economics literature [28] and is often employed by respected ethicists [29]. However, the fair market value of a kidney from a living donor is not known because the National Organ Transplant Act prohibits the buying and selling of human organs for transplantation.

To estimate the fair market value (see S1 File), we begin with the fact that Medicare pays organ procurement organizations (OPOs) about $55,000 for a kidney from a deceased donor [7]. But a kidney from a living donor is worth more than one from a deceased donor because the expected functional life of a graft from a living donor (for a person on the kidney waiting list) is about 43% longer [8]. This alone would boost the value of a living donor kidney by $23,650. In addition, when a kidney transplant fails, a patient typically incurs costs totaling about $233,000 ($88,000 for the cost of graft failure plus $145,000 for a second kidney transplant [8]). For a living donor kidney, this cost occurs 4.2 years later than for a deceased donor kidney. So using a real discount rate of 3%, the delay in incurring this expense makes a kidney from a living donor more valuable by $20,665.

On the other hand, it costs about $20,000 to remove a kidney from a living donor [30] and about $5,000 for the tests of the donor beforehand, both of which reduce the value of a donated kidney. So taking all of these factors into consideration, the estimated value of a kidney from a living donor is roughly $75,000 ($55,000 + $23,650 + $20,665 - $25,000 = $74,315).

This is the amount the government would initially offer for a kidney from a living donor. Over time, the government would likely adjust this amount to balance the quantity of kidneys supplied and demanded. In any event, we will see below that our conclusions do not depend crucially on the exact magnitude of this value because it is compared to the benefit that poor kidney recipients receive from a transplant, which is more than an order of magnitude larger.

Thus, at the current time when donor compensation is prohibited, a living donor is paid nothing for a kidney that has a value of about $75,000, so the amount of exploitation (as we have defined it) is $75,000 (**Table 1**, row 4). On the other hand, if government compensation of living kidney donors were increased to $75,000, exploitation would fall to zero (**Table 1**, row 5). If compensation were increased to $100,000, exploitation would decline to a negative $25,000, i.e., the donor would receive a net benefit of $25,000 (**Table 1**, row 6). Thus, exploitation can be reduced, eliminated, or turned into a net benefit by increasing the amount of compensation.

**Table 1:** Summary: Definitions and preliminary results.

	Transplants per recipient	Present value of kidney transplantation to a recipient (over a lifetime)	
**If donors are not compensated** (resulting in a kidney shortage)	1	$937,000	
**If donors are compensated the fair market value of a kidney** (ending the kidney shortage)	2	$1,330,000	
**Estimated value of a kidney from a living donor**	$75,000		
**Relationship between compensation and exploitation**	**Compensation** (per kidney)	**Exploitation** (per kidney) (exploitation = $75,000 minus compensation)	
	$0	$75,000	
	$75,000	$0	
	$100,000	- $25,000	
**Definitions**			
Exploitation	Paying less than the fair market value of a good or service		
The Poor	Adults who have not graduated from high school (12% of U.S. population)		
**Yearly kidney failure statistics in the U.S. (2016)**			
Patients newly diagnosed with chronic kidney failure		125,399	
Added to kidney transplant wait list		30,869	
Removed from kidney transplant wait list		33,291	
Transplanted using kidney from deceased donor		13,501	
Transplanted using kidney from living donor		5,335	
Died while on wait list		4,830	
Became too sick to transplant		4,411	
Other		5,214	

Indeed, if policymakers conclude (or pilot studies suggest) an even greater sum is needed to ensure that donors are not exploited, the government could easily afford to increase compensation above $75,000. The savings from stopping dialysis after transplantation are so great that compensation could be increased to as much as $320,000 per kidney [8] before the taxpayer would no longer save money by having the government compensate donors.

It is clear that exploitation of kidney donors, as we have defined it, applies to kidney donors of all income levels. However, since the distinguished critics of donor compensation focus on the exploitation of the poor, we will address that criticism.

Data on the income levels of kidney donors and recipients is not readily available, but data on educational level, a recognized proxy for income (see S4 File), is available for kidney recipients. Therefore, we will define the poor as adults who have not graduated from high school, which is about 12% of the U.S. population. (According to the U.S. Census Bureau, 13.5% of the U.S. population lived in poverty in 2015 [31].)

In addition, we present data showing poor patients are: (a) less likely to qualify for (or be assigned to) the kidney transplant waiting list and receive a transplant from a deceased donor, and (b) less likely to receive a transplant from a living donor.

## Results

**Fig 1:**
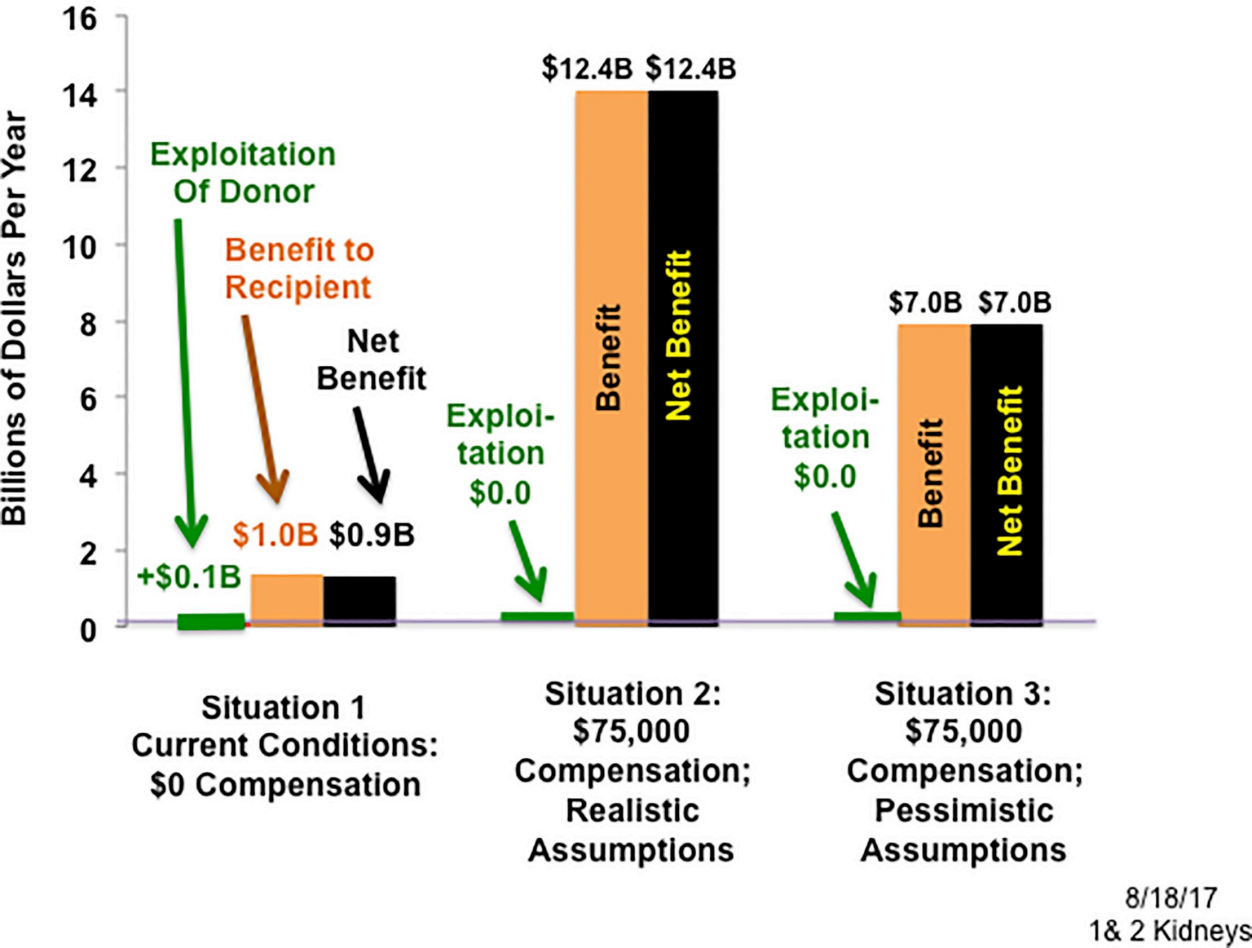
Exploitation, benefit, and net benefit for the poor: With donor compensation of $0 and $75,000.

Given these definitions and preliminary calculations, the exploitation of poor kidney donors was compared with the benefit that poor kidney recipients receive from a transplant to estimate the net effect on the poor in three different situations. The first is the current situation in the U.S. where compensation of kidney donors is prohibited. In the second, the government compensates living kidney donors the $75,000 fair market value of a kidney under realistic (but conservative) assumptions about the response of poor donors and recipients. The third situation is a sensitivity analysis to show how robust the conclusions of Situation 2 are—the government again compensates living kidney donors $75,000, but under assumptions that are very pessimistic for the welfare of the poor. **Table 2** and **Fig 1** summarize the calculations and results for the three situations.

**Table 2:** Net benefit to the poor (per year) if donors are compensated $0 or $75,000.

(1)	(2)	(3)	(4)	(5)	(6) = (3) X (4) X value to a recipient of 1 or 2 transplants	(7) = (3) X (5) X [$75,000 - (2)]	(8) = (6)—(7)
Situation	Assumptions				Aggregate results (per year)		
	Govern-ment compen-sation per donor	Trans-plants per year	Recip-ients who are poor	Donors who are poor	Benefit to poor recipients	Exploi-tation of poor donors	Net benefit to the poor
**Situation 1:** **Current conditions in the U.S. $0 donor compensation**	$0	17,500	6%	4%	$1.0B = 17,500 X 6% X $937,000 for 1 transplant	$0.1B = 17,500 X 4% X [$75,000 - $0] for 1 transplant	$0.9B = $1.0B - $0.1B
**Situation 2:** **$75,000 donor compensation under realistic assumptions**	$75,000	31,000	30%	50%	$12.4B = 31,000 X 30% X $1,330,000 for 2 transplants	$0 = 31,000 X 50% X 2 X [$75,000 - $75,000] for 2 transplants	$12.4B = $12.4B - $0
**Situation 3:** **$75,000 donor compensation under pessimistic assumptions**	$75,000	31,000	17%	100%	$7.0B = 31,000 X 17% X $1,330,000 for 2 transplants	$0 = 31,000 X 100% X 2 X [$75,000 - $75,000] for 2 transplants	$7.0B = $7.0B - $0

### Situation 1: Current conditions in the U.S.

- Compensation of kidney donors is prohibited,
- Partly as a result, only about 17,500 transplants take place each year (using kidneys from both living and deceased donors),
- The poor receive only 6% of these transplants [32] (much less than their 12% share of the general population and their 34% share [33] of patients newly diagnosed with ESRD),
- The poor provide an estimated 4% of living donor kidneys (close to the 3% share of living donor kidneys they receive) [32].

Since donor compensation is prohibited, the exploitation of an individual living kidney donor is the maximum amount: $75,000. And if we make the conservative simplifying assumption that the level of exploitation of deceased kidney donors is the same as for living donors (see S2 File), then the aggregate exploitation of all poor kidney donors is $0.1B/yr. [= 17,500 transplants/yr. X 4% of transplant kidneys donated by the poor X exploitation of $75,000 per donor]. Nonetheless, even this slight over-estimate is still an order of magnitude less than the aggregate benefit to all poor kidney recipients, which is $1.0B/yr. [= 17,500 transplants/yr. X 6% of transplant kidneys received by the poor X $937,000 benefit per recipient from one transplant]. Therefore, under current conditions, the net benefit to the poor as a group from transplantation is $0.9B/yr. (See **Table 2**, row 1, columns 6–8.)

### Situation 2: Donor compensation of $75,000 under realistic assumptions

- Now assume the government increases the compensation of living kidney donors from zero to the $75,000 fair market value of a kidney.
- This causes the number of kidney transplants to increase to more than 31,000 per year (enough to supply the yearly additions to the kidney transplant waiting list).
- With the kidney shortage ended, poor patients would receive 30% of these kidney transplants (only slightly less than their 34% share of the ESRD population as explained in the Discussion section).
- The poor provide 50% of living donor kidneys (more than four times their 12% share of the general population).

Then the exploitation of individual kidney donors would be zero (since donors would be compensated for the fair-market value of their kidney). So the aggregate exploitation of all poor donors would also be zero, while the aggregate benefit to poor kidney recipients would be $12.4B/yr. [= 31,000 transplants/yr. X 30% of transplant kidneys received by the poor X $1,330,000 benefit per recipient from two transplants]. Therefore, the net benefit to the poor as a group would also be $12.4B/yr. This is about 13 times the $0.9B/yr. net benefit to the poor in the current situation.

It is clear from **Table 2** and **Fig 1** that the conclusions of Situation 2 are robust. The estimated value of kidney transplantation to a poor kidney recipient is so large ($1,330,000), and the purported exploitation of a poor kidney donor is so small (zero to $75,000), that any reasonable alternative assumptions would likely yield the same conclusion—that the poor as a group would be far better off if donors were compensated.

### Situation 3: Donor compensation of $75,000 under pessimistic assumptions

This point is illustrated by the following sensitivity analysis in which the assumptions are deliberately skewed to produce a result unfavorable to the poor.

(a) The government again compensates kidney donors $75,000, and
(b) This again results in more than 31,000 kidney transplants per year.

But now, for purposes of illustration only, the following pessimistic assumptions are made:

(c) The poor provide 100% of the donor kidneys, but
(d) They receive only 17% of kidney transplants (half of their 34% share of the kidney failure population).

Then under these pessimistic assumptions for the welfare of the poor, the aggregate exploitation of poor kidney donors would again be zero, and the aggregate benefit to poor kidney recipients would be $7.0B/yr. [= 31,000 transplants/yr. X 17% of transplant kidneys received by the poor X $1,330,000 benefit per recipient from two transplants]. Therefore, the net benefit to the poor from transplantation would also be $7.0B/yr., which is seven times the $0.9B/yr. net benefit in the current situation.

### Other ways government compensation would affect the poor

Government compensation of living kidney donors would also alleviate two other serious problems currently facing poor patients in need of a transplant. These patients are: (a) less likely to be added to the kidney transplant waiting list and receive a transplant from a deceased donor, and (b) less likely to receive a transplant from a living donor.

**Fig 2:**
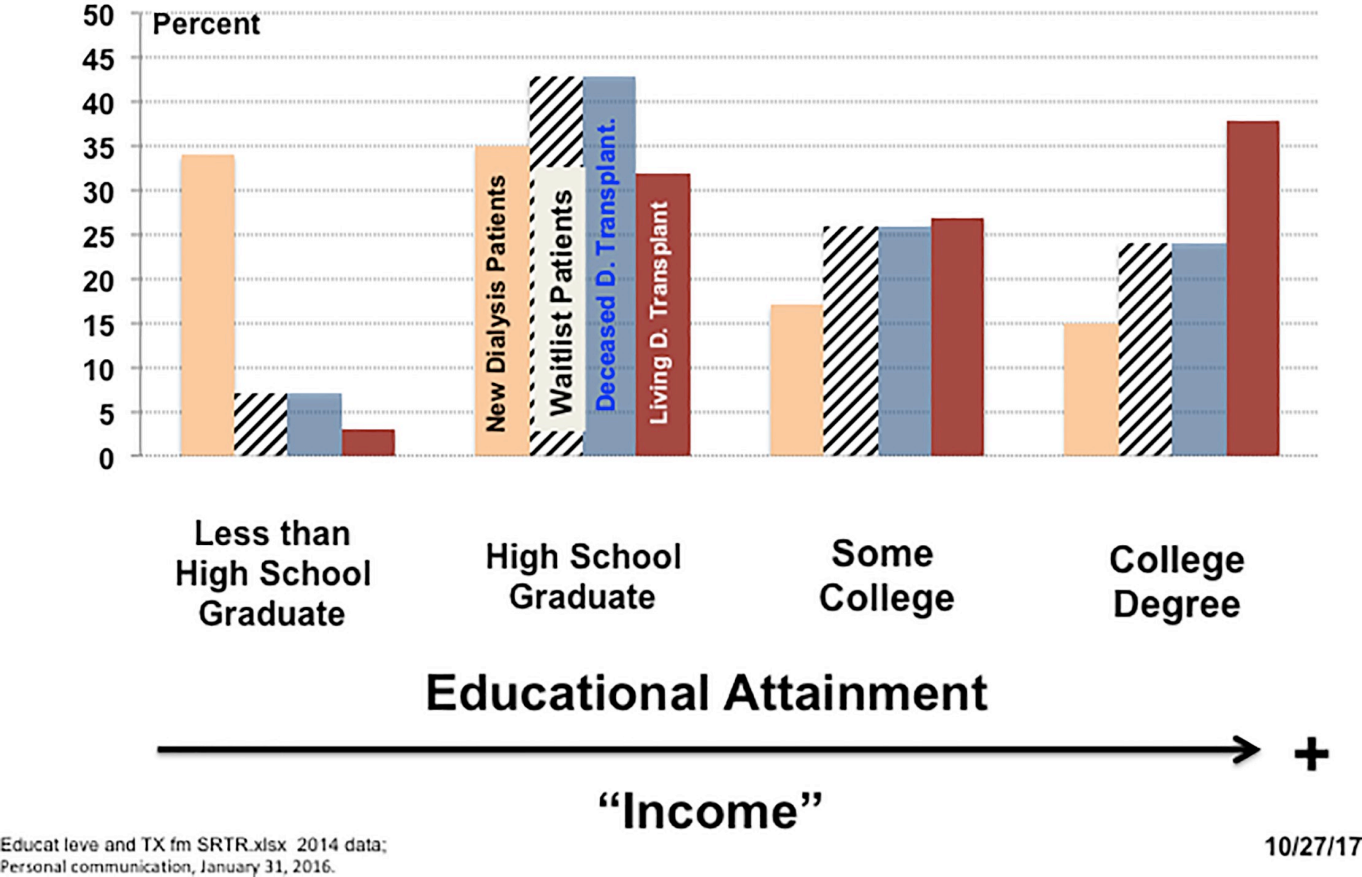
Percent of new dialysis, waitlist, and transplanted patients at each educational level.

These two problems are illustrated in **Fig 2**, using data from a census of all U.S. kidney transplants in 2014 (SRTR [33]). The columns indicate the percent of different patient groups who have attained four different levels of education: (a) less than high school graduate, (b) high school graduate, (c) attended some college (but no degree), and (d) college degree (associate, bachelors, or graduate).

For instance, the tan columns show that, among patients starting dialysis, 33% have not finished high school, 35% are high school graduates, 17% have some college, and 15% have college degrees (percentages sum to 100%). Patients starting dialysis are concentrated in the lowest two educational groups.

The hatched columns indicate the percent of new dialysis patients who are added to the kidney waiting list. Note that only 7% are not high school graduates. Similarly, the blue columns show the percent of new dialysis patients who receive a transplant from a *deceased* donor; again only 7% are not high school graduates.

The red columns indicate the percent of new dialysis patients who receive a transplant from a *living* donor; an even smaller 3% are not high school graduates.

Thus, even though the least educated (poorest) patients are over-represented among those diagnosed with ESRD, they are under-represented on the kidney transplant waiting list and among those who receive a kidney from either a deceased or living donor.

## Discussion

### The barriers the poor face in obtaining transplants

**Fig 2** clearly indicates the biggest barrier poor patients face in obtaining a deceased donor kidney is being placed on the waiting list, not getting a transplant once on the list (where the current system seems to work reasonably well in allocating deceased donor kidneys to the poor). This wait list admission problem does not necessarily reflect deliberate discrimination against the poor. Instead, given the severe kidney shortage under the current system, admission to the waiting list must be limited by some criteria, and these criteria favor the healthier candidates with the best prospects for a successful transplant—who happen to be the more affluent.

With regard to kidneys from living donors, the problem for poor recipients is that most of their relatives and friends are also poor and hence less able to bear the burden of being a living donor (lost wages, etc.).

Both barriers for the poor could be circumvented by government compensation of kidney donors because that would end the kidney shortage. Enough kidneys would be available for all who needed one, rich or poor.

Note, however, that even if the kidney shortage is ended, poor patients may not receive kidney transplants in full proportion to their 34% share of the ESRD population because of other factors. For example, many non-elderly Medicare patients lose all Medicare coverage including immunosuppressive therapy 36 months after a transplant [34]. That policy creates an incentive for patients, particularly the poor and young, to not even apply for a transplant. Also, the poor are more likely to live in rural areas, far from a transplant center, making it difficult for them to travel to a center to apply for the waiting list or to receive a transplant.

The opponents and proponents of donor compensation have very different perspectives on the relation between compensation and exploitation. Opponents usually cite the conditions in illegal markets (commonly referred to as “black” markets) for kidneys in poor countries as evidence that compensation leads to exploitation [27,35]. Proponents usually refer to a proposed legal allocation process in the U.S. that would be controlled by the government and would be specifically designed to prevent exploitation [3,8,12,36]. **Table 3** summarizes the differences between the two views. Compensation for living donors would be paid in a delayed non-cash form—such as tax credits, health insurance, tuition assistance, retirement funds, etc.—so people who are desperate for cash would not be tempted to sell a kidney. Before adopting this proposal, the government should sponsor pilot programs to test the various features and discover any unintended consequences (see S3 File for the details of our specific proposal).

**Table 3:** Comparing illegal markets in poor countries with a proposed legal regulated allocation process in the U.S.

Illegal markets in poor countries	Proposed legal regulated allocation process in U.S.
Organized by criminals	Run by government and medical professionals
No laws to protect buyers and sellers	Large body of law to protect buyers and sellers
No courts to settle disputes	Courts can be readily accessed
No advertising of prices or other information	Ratings of medical institutions readily available
Little government supervision	Government controls both sides of market
Opportunistic focus on transplant tourists	Intended to end kidney shortage and save lives of U.S. citizens, and end attraction of illegal markets
Screening of donors is perfunctory	Donors are carefully screened
Donors are poor, illiterate, and easily misled	Donors are fully advised of all risks and provide valid, informed consent
Unhygienic medical facilities	World class medical facilities
Immediate cash payment	Compensation in a non-cash delayed form
Little post-operative care	Insurance policy against any future medical problems
Rich buy kidneys from the poor	Organs allocated fairly to all income levels
Donors are subject to commodification and objectification	Donors treated with concern, respect, and dignity
Donors and recipients recruited from foreign countries	Donors and recipients are all from U.S. No seeking lower-compensated donors outside U.S.
Scientific research not possible or of interest	Clinical trials and comparative effectiveness research to determine best practices

### Limitations of the study

Many ethical concerns have been raised about government compensation of kidney donors. In this paper, we have chosen to focus on just one of them—that donor compensation would exploit the poor—because (a) that is the main argument made by many distinguished opponents of donor compensation, and (b) that hypothesis can be empirically tested.

Oxford ethicist Janet Radcliffe Richards has also extensively critiqued the exploitation argument [37]. With regard to the contention that poor people would not donate a kidney if they had enough money, she points out that this reasoning would apply to much paid work. This reasoning does not differentiate organ selling from innumerable other activities (e.g., rubbish removal, clearing sewers, etc.) that people would not do if they had enough money. Also, if we say the rich are exploiting poor donors, then, according to Richards, we can equally say that all persons in a weaker position are being exploited by people in a stronger position. For example, those who supply a kidney in return for compensation can be said to be exploiting those who are in desperate need of a transplant kidney.

Some of the other ethical concerns about compensating kidney donors have been expertly addressed by other researchers:

Nobel Laureate Alvin Roth [38] wrote the seminal article on this subject, noting that some transactions are not repugnant as gifts or in-kind exchanges, but become repugnant when money is involved. These transactions are considered repugnant because they (a) treat people like objects, (b) could be coercive, leaving some poor people open to exploitation, and (c) could lead to a slippery slope of even more repugnant transactions.

Sandro Ambuehl, Muriel Niederle, and Alvin Roth [39] surveyed the general public to see how their views on the ethical appropriateness of paid participation in medical experiments changed as the level of compensation changed. They found that the public generally thought some form of payment was more ethical than purely voluntary participation, and that in-kind compensation was most ethical. But some participants thought very high amounts of compensation were less ethical.

Julio Elías, Nicola Lacetera, and Mario Macis [40] found that support for a market-based solution to the organ shortage increased when people were presented with documented and verifiable information about its potential benefits. This shows that empirical evidence can affect what society considers to be ethically acceptable.

Sandro Ambuehl and Axel Ockenfels [41] in a survey about human egg donation showed that when the acquisition of information is costly, individuals with higher marginal costs of information often respond more to higher compensation. Thus, as compensation increases, people who find it more difficult to be well informed comprise an increasing fraction of participants. Consequently, policy makers should go to some lengths to ensure participants have a thorough understanding of possible negative outcomes.

**Undue inducement is a term originating in law which means improper influence that deprives a person of freedom of choice or substitutes another's choice or desire for the person's own (Merriam-Webster Dictionary)**. **In an article describing persons interviewed on the regional rail and urban trolley lines in Philadelphia, Halpern et al. [42] “… found no evidence that any of the 3 main concerns with a regulated system of payments for living kidney donation would manifest if such a market were established. Providing payments did not dull persons’ sensitivity to the risks associated with donor nephrectomy, suggesting that payment does not represent an undue inducement—one that would make rational choice difficult. Furthermore, providing payments did not preferentially motivate poorer persons to sell a kidney, suggesting that payment does not represent an unjust inducement—one that would put substantially more pressure on poorer persons than on wealthier persons.”**

Also, Gordon et al. [43] say there is a range of compensation between beginning to consider donation and beginning to feel undue inducement to donate, suggesting leeway for offering acceptable amounts of financial compensation before exerting an undue inducement on people to donate.

**Fisher et al**. **[44] say: “Despite repeated calls for a pilot study to assess the impact of financial compensation on living kidney donation rates, many fear that financial incentives will exploit vulnerable individuals and cast the field of transplantation in a negative public light, ultimately reducing donation rates.”**

Note that we took many of these ethical concerns into account when designing (a) our proposed legal regulated allocation process in the U.S. in **Table** 3, and (b) our specific proposal to compensate kidney donors in the S3 File.

#### There are some limitations to the analyses and values calculated in this study

This paper used educational attainment as a proxy for income. A great deal of research shows a close correlation between the two (see S4 File). To establish the fair market value of a kidney from a living donor, estimates from published research were used. Actual market prices would be preferred but are unavailable due to a federal law that prohibits the selling of human organs for transplantation.

Nothing in this paper should be construed as advocating that poor people should accept compensation for their kidneys. Indeed, if the poor were completely prohibited from accepting compensation—a simple policy alternative—it would just strengthen the case for our basic conclusion that government compensation of living kidney donors would greatly benefit the poor. Poor kidney recipients would still receive the benefits of transplantation, but there would be no chance poor donors might be exploited.

## Conclusion

The purpose of this study was to empirically test the hypothesis that government compensation of kidney donors would exploit the poor. We concluded that if the government rewards living kidney donors with a package of non-cash benefits worth about $75,000 per kidney, donors would not be exploited.

Much more important, compensation would likely end the kidney shortage, enabling many more patients with kidney failure to obtain transplants and live longer and healthier lives. The value of transplantation to a U.S. recipient is about $1,330,000, which is an order of magnitude greater than any supposed exploitation of living kidney donors (zero to $75,000 per donor). Indeed, compensating kidney donors would increase the aggregate benefit to the poor from transplantation about 13-fold to $12.4 billion per year from $0.9 billion currently.

Note this is not a case of one group of poor people benefiting from government compensation of kidney donors while another group of poor people is made worse off. Rather, poor kidney recipients are greatly benefited, but poor kidney donors are no worse off because they are compensated for the fair market value of their kidney. Moreover, if the government chooses to compensate kidney donors more than $75,000—which it could easily afford to do and still save money for the taxpayer—it could ensure poor donors would also be better off, i.e., they would also receive a net benefit.

## Supporting information

S1 File Estimating the value of a kidney from a living donor. (PDF)

S2 File Reasons for focusing on a living donor. (PDF)

S3 File Our specific proposal to compensate kidney donors. (PDF)

S4 File Employing educational attainment as a proxy for income. (PDF)
