# Supplementary material for: Would government compensation of living kidney donors exploit the poor? An empirical analysis
Source: PLoS One. 2018 Nov 28;13(11):e0205655. doi: 10.1371/journal.pone.0205655 (PMC6261427; doi:10.1371/journal.pone.0205655)
Supplement: S1 File — (PDF) [file pone.0205655.s001.pdf]

S 1

Would government compensation of living kidney donors exploit the poor?

Held, McCormick, Chertow, Peters, and Roberts.

Supplement 1 (S 1) : Details of Estimating the Value of a Kidney from a Living Donor

1. Beard ([7] p. 81) estimates Medicare is willing to pay
Organ Procurement Organizations $50,000-$60,000 for a kidney
from a *deceased* donor
$55,000

2. According to Held-McCormick [8, Supplement 12,
Table S12-1, line 1], for patients on the kidney waiting list, living
donor kidneys last 1.43 (= 14.2/9.9) times longer than deceased
donor kidneys. So that factor alone should boost the value of a living
donor kidney to ($55,000 X 1.43) =
$78,650

3. In addition, according to to Held-McCormick [8, p. 879], it costs
$88,000 when a graft fails, plus $145,000 for a second kidney, for
a total expense of $233,000. For kidneys from deceased donors, this
expense occurs on average after 9.3 years; for kidneys from living
donors, after 13.5 years. So, using a real discount rate of 3%, the
discounted present value of this cost for kidneys from deceased donors
is 233,000/($1.03^{9.3}$) = $176,999. And the discounted present value of
kidneys from living donors is 233,000/($1.03^{13.5}$) = $156,334. So the
delay in incurring this expense is a second reason that kidneys from
living donors are more valuable by $176,999 - $156,334 =
+$20,665

4. So the value of a kidney from a living donor is
$78,650 + $20,665 =
$99,315

5. But it cost $25,000 to test living donors and perform a nephrectomy [26]. -
$25,000

6. So a living kidney donor is currently asked to give up a kidney with a value of about $74,315 (= $99,315 - $25,000) and receive nothing in return. So the level of exploitation of a living donor is
$74,315

7. Which in our examples we conservatively round up to $75,000
