# Supplementary material for: Would government compensation of living kidney donors exploit the poor? An empirical analysis
Source: PLoS One. 2018 Nov 28;13(11):e0205655. doi: 10.1371/journal.pone.0205655 (PMC6261427; doi:10.1371/journal.pone.0205655)
Supplement: S2 File — (PDF) [file pone.0205655.s002.pdf]

S 2

Would government compensation of living kidney donors exploit the poor?

Held, McCormick, Chertow, Peters, and Roberts.

Supplement 2 (S 2) : Reasons for Focusing on Living Donors

This study focused on kidneys from living rather than deceased donors because there are not enough of the latter to meet the current and future demand for kidney transplants. According to the 2015 SRTR-OPTN Annual Data Report [2] the 11,000 deceased donor kidney organs transplanted each year represent about 70 percent of eligible deaths (i.e., deaths under circumstances that enable the kidneys to be transplanted). This implies a maximum of 16,000 kidneys per year might be recovered from deceased donors. The additional 5,000 kidneys would be helpful, but the 16,000 total is far short of the estimated 43,000 transplant kidneys per year needed to reduce the waiting list to zero over five years, or the 35,000 per year needed to keep the waiting list at zero thereafter [8]. In addition, if there were no longer a severe kidney shortage, many more of the 125,000 patients diagnosed with ESRD each year might be admitted to the kidney transplant waiting list, increasing the future demand for transplant kidneys significantly above 35,000 per year.

Since almost all of the additional kidneys to meet future demand will have to come from living donors, we made the conservative simplifying assumption that the exploitation of deceased kidney donors was the same as for living donors -- $75,000 -- although in reality it is likely less. This assumption is conservative because it boosted the aggregate exploitation of poor kidney donors in Situation 1 to $0.052 billion (rounded up to $0.1 billion). Yet this is still an order of

magnitude smaller than the $1.0 billion aggregate benefit to poor kidney recipients, which is the key result of Situation 1.

If instead we had calculated the value of a deceased donor kidney separately and multiplied it by the number of deceased donor transplants, it would have significantly complicated the analysis with essentially no effect on our results. For example, dividing the 17,500 total transplants into 5,250 from living donors and 12,250 from deceased donors (which is very close to their actual numbers in 2015 -- 5,331 and 12,280 -- as shown in Table 1), and arbitrarily assuming the exploitation of a deceased donor is only half that of a living donor, then the aggregate amount of exploitation in Situation 1 would be:

Living donors: 5,250 X $75,000 X 0.04	= $0.016 billion
Deceased donors: 12,250 X $37,500 X 0.04	= $0.018 billion
Total	= $0.034 billion.

This is slightly less than our calculation of $0.052 billion in the main text, but both are more than an order of magnitude less than the $1.0 billion aggregate benefit to poor kidney recipients, the key result of Situation 1.

This simplifying assumption also conservatively allows for the possibility in Situation 2 that, with an ample supply of the superior living donor kidneys available, surgeons and patients may elect to use only living donor kidneys.
