# Supplementary material for: Would government compensation of living kidney donors exploit the poor? An empirical analysis
Source: PLoS One. 2018 Nov 28;13(11):e0205655. doi: 10.1371/journal.pone.0205655 (PMC6261427; doi:10.1371/journal.pone.0205655)
Supplement: S3 File — (PDF) [file pone.0205655.s003.pdf]

S 3

Would government compensation of living kidney donors exploit the poor?

Held, McCormick, Chertow, Peters, and Roberts.

Supplement 3 (S 3) : Our Specific Proposal to Compensate Kidney Donor

Our specific proposal for a legal regulated allocation process in the U.S. is to have the government (not private individuals) compensate living kidney donors $75,000 (and deceased donors somewhat less) to ensure donors are not exploited, nor subjected to undue influence or coercion. With experience, the government would likely adjust these numbers to balance supply and demand. This compensation would be considered an expression of appreciation by society for someone who has given the gift of life to another. It would include an insurance policy against any health problems that might develop in the future as a result of the donation, including disability and death. Compensation for living donors would be paid in a delayed non-cash form -- such as tax credits, health insurance, tuition assistance, retirement funds, etc. -- so people who are desperate for cash would not be tempted to sell a kidney. Compensation for *deceased* donors would be paid to their estate. All other aspects of the kidney procurement and allocation process would continue as they are under the current system. Except education of living donors about the possible hazards associated with kidney donation would be much more thorough than it is now and would include specific probabilities of bad outcomes. Kidneys would be allocated as the organs from deceased donors are now—by the federally funded and managed Organ Procurement and Transplant Network (currently administered under contract by United Network for Organ Sharing). Before adopting this proposal, the government should sponsor pilot programs to test the various features and discover any unintended consequences.

Note that this proposal is far from a free market in kidneys. Rather, it is designed to address many of the ethical concerns discussed in the Limitations of the Study section and elsewhere.
