# Supplementary material for: Would government compensation of living kidney donors exploit the poor? An empirical analysis
Source: PLoS One. 2018 Nov 28;13(11):e0205655. doi: 10.1371/journal.pone.0205655 (PMC6261427; doi:10.1371/journal.pone.0205655)
Supplement: S4 File — (PDF) [file pone.0205655.s004.pdf]

S4: Supplement 4

Would government compensation of living kidney donors exploit the poor?

Held, McCormick, Chertow, Peters, and Roberts.

Supplement 4 (S 4): S 4 (Employing educational attainment as a proxy for income).

This research used educational attainment as a proxy for income. Much research has shown a close correlation between the two. See Figure S4-1 for an overview of this association. Education is also related to the unemployment rate, also shown in Figure S4-2.

Figure S4-1

Unemployment rates and earnings by educational attainment, 2017

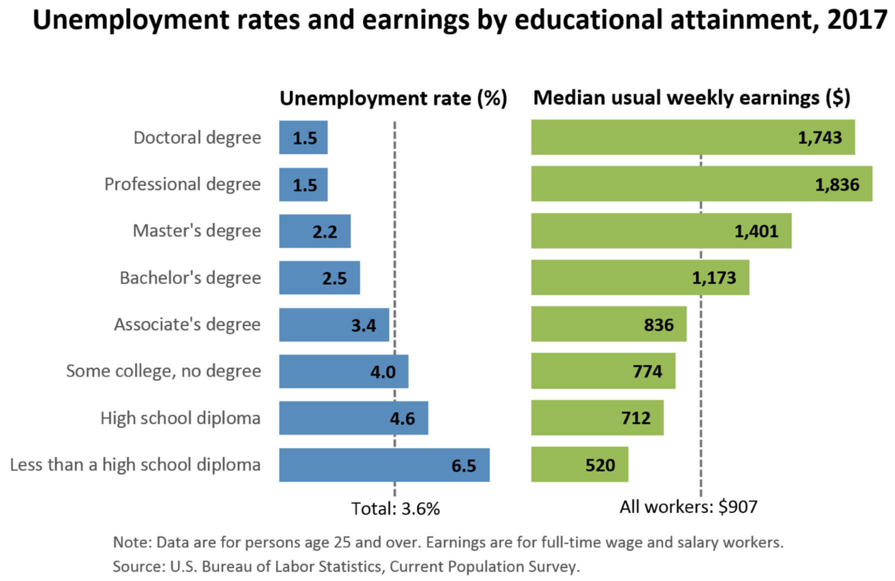

https://www.bls.gov/emp/chart-unemployment-earnings-education.htm

Figure S4-2
Median incomes by educational attainment

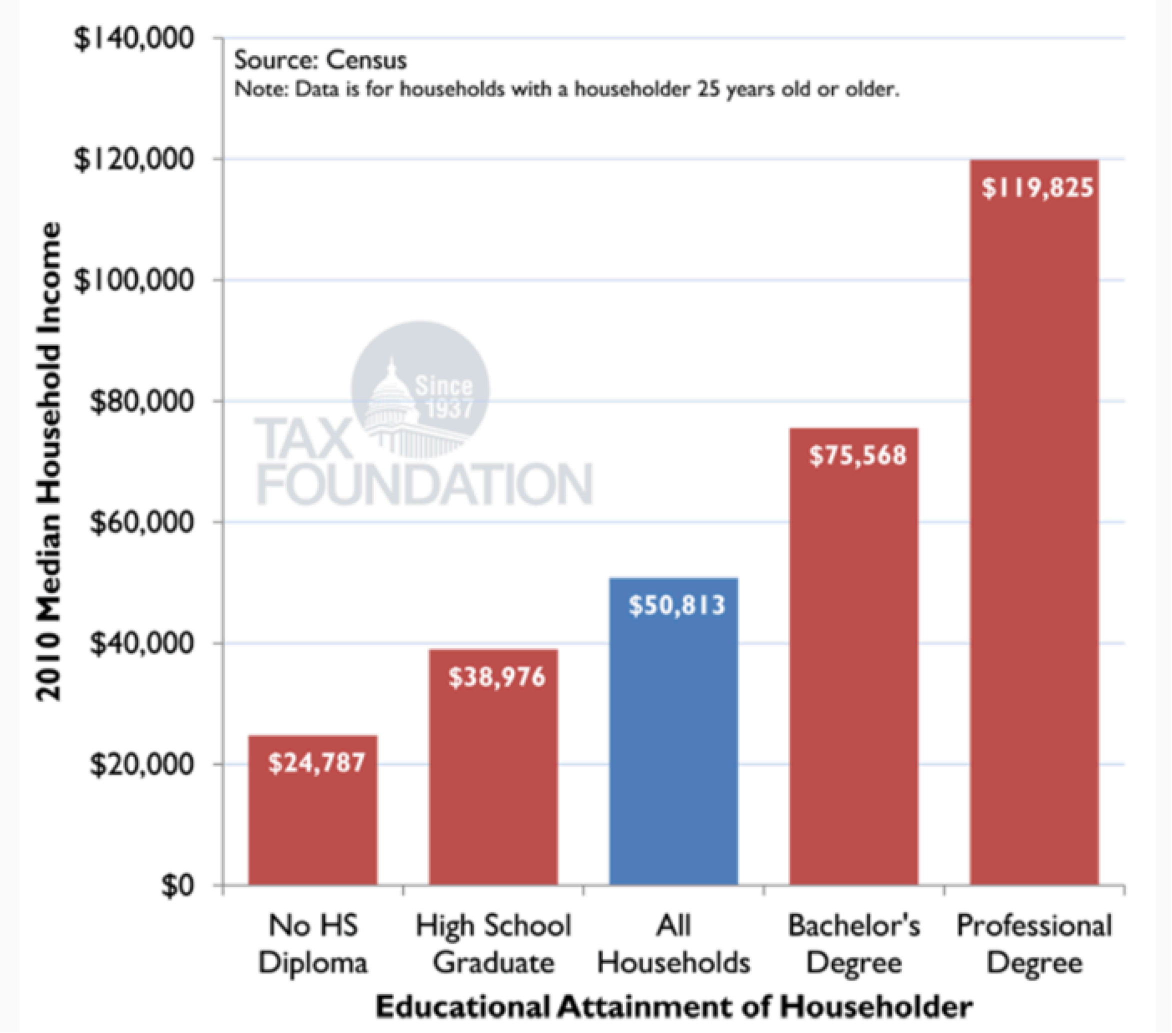

https://taxfoundation.org/chart-day-income-levels-vs-education-levels
